# Suggestive Therapeutics in Psychopathia Sexualis

**Published:** 1895-03

**Authors:** 


### Suggestive Therapeutics in Psychopathia Sexualis. By Dr. A. Schrencknotzing, of Munich. Three hundred and twenty-five pages. Price, $2.50, in cloth. Sold only by subscription. F. A. Davis Co., 1914–16 Cherry Street, Philadelphia, Pa.

This book is a companion volume to the now famous treatise, *Psycopathia Sexualis*, of Dr. R. von Krafft-Ebing, which was reviewed in the pages of this journal in September, 1893, page 493.

It is not yet issued from the press, but will appear shortly, and will be duly reviewed.

